# Oligosaccharides from *Morinda officinalis* Slow the Progress of Aging Mice by Regulating the Key Microbiota-Metabolite Pairs

**DOI:** 10.1155/2019/9306834

**Published:** 2019-12-19

**Authors:** Yang Xin, Chen Diling, Chen Tianlu, Zhao Jun, Tang Xiaocui, Guo Yinrui, Shuai Ou, Deng Tianming, Hu Guoyan

**Affiliations:** ^1^Department of Pharmacy, The Fifth Affiliated Hospital of Guangzhou Medical University, Guangzhou 510700, China; ^2^State Key Laboratory of Applied Microbiology Southern China, Guangdong Provincial Key Laboratory of Microbial Culture Collection and Application, Guangdong Open Laboratory of Applied Microbiology, Guangdong Institute of Microbiology, Guangdong Academy of Sciences, Guangzhou 510070, China; ^3^Shanghai Key Laboratory of Diabetes Mellitus and Center for Translational Medicine, Shanghai Jiao Tong University Affiliated Sixth People's Hospital, Shanghai 200233, China; ^4^Department of Obstetrics and Gynecology, Guangdong Women and Children Hospital, Guangzhou 510010, China; ^5^Department of Geriatrics, The Fifth Affiliated Hospital of Guangzhou Medical University, Guangzhou 510700, China; ^6^Department of Clinical Laboratory, The Fifth Affiliated Hospital of Guangzhou Medical University, Guangzhou 510700, China

## Abstract

The gut microbiota is considered an important factor in the progression of Alzheimer's disease (AD). Active research on the association between the metabolome and the gut microbiome is ongoing and can provide a large amount of beneficial information about the interactions between the microbiome and the metabolome. Previous studies have shown that the oligosaccharides from *Morinda officinalis* (OMO) can delay the progress of AD in model animals by regulating the diversity of the gut microbiome and metabolic components, and the correlation between the gut microbiome and metabolic components still needs to be further verified. This study applied a new two-level strategy to investigate and ensure the accuracy and consistency of the results. This strategy can be used to determine the association between the gut microbiome and serum metabolome in APP/PS1 transgenic mice and C57BL/6J male mice. The “*4C0d-2* spp.-Cholesterol,” “*CW040* spp.-L-valine,” “*CW040* spp.-L-acetylcarnitine,” “*RF39* spp.-L-valine,” “*TM7-3* spp.-L-valine,” and “*TM7-3* spp.-L-acetylcarnitine” associations among specific “microbiota-metabolite” pairs were further identified based on univariate and multivariate correlation analyses and functional analyses. The key relevant pairs were verified by an independent oligosaccharide intervention study, and the gut microbiome and serum metabolome of the OMO intervention group were similar to those of the normal group. The results indicate that OMO can significantly suppress Alzheimer's disease by regulating the key microbiota-metabolite pairs. Therefore, this two-level strategy is effective in identifying the principal correlations in large datasets obtained from combinations of multiomic studies and further enhancing our understanding of the correlation between the brain and gut in patients with AD.

## 1. Introduction

Alzheimer's disease (AD) is a neurodegenerative disease that will affect nearly 82 million people by 2030 and 152 million people by 2050 [1], according to the 2018 World AD Congress Report. China has become the country with the largest number of AD patients in the world. At present, the incidence of AD has reached more than 8 million [2], and the incidence of AD in elderly individuals over 65 years old is 4%∼6% [3], which is increasing. The cost of AD treatment would be a very large financial burden, and the public's awareness of AD is severely inadequate [4]. The majority of patients with AD are not effectively diagnosed or treated. Therefore, the identification of markers and signaling pathways leading to AD is essential for understanding the pathogenesis of this disease and its effective treatment.

According to recent research, multiple pathological characteristics of AD are related to gut microbial infections [5–7]. The microbiome-gut-brain axis connects nerve, hormone, and immune signals between the gut and the brain, which has profound effects on the growth and health of mammals [8]. The gut microbiota is considered an important factor for determining the progression of AD [9–11]. However, the mechanism of this relationship remains unclear. Thus, in-depth studies are required to verify the existing routes of bidirectional communication [12] to elucidate the relevant molecular mechanisms.

In the early stage, we proposed a new treatment strategy to reverse the microbiological maladjustment related to the pathological state by improving the gut microbiome of animals with AD [13]. Oligosaccharides isolated from *Morinda officinalis* (OMO) could improve the memory ability of rats with beta-amyloid-induced dementia in the water maze test [14, 15]. The SOD, CAT, and GSH-Px activities were high, and the MDA content was lowered in the brain tissue of rats with dementia, as determined by kit-based detection. In addition, the acetylcholinesterase levels decreased, the acetylcholine levels and brain energy metabolism levels increased, and Na^+^/K^+^-ATPase activity increased, indicating that OMO can significantly improve dementia symptoms in animal models of AD [16, 17]. Bajijiasu, one of the main components of OMO, is a neuroprotective agent that plays a neurotoxic protective role in A*β*_25-35_-induced PC12 cells and the APP/PS1 mouse model [17–19]. This OMO component may act through antioxidant, anti-inflammatory, and neurotransmitter mechanisms to improve AD symptoms.

Furthermore, OMO can improve the function of the gut microbiota to inhibit the progression of AD [13, 20, 21]. We studied the changes in the gut microbiome and serum metabolite levels in animal models of AD [13]. Based on previous studies, it was found through bioinformatics-based mining that OMO can delay the progress of AD by regulating the key “microbiome-metabolite” pair, and preliminary data on microbiome-metabolite interactions were established. This study expands our understanding of the microbiome-gut-brain axis, as the metabolome and particularly some of the newly discovered serum metabolites may serve as the endpoints of new pathways connecting the gut to the brain.

In this study, the APPswe/PS1dE9 (APP/PS1) transgenic mouse model [22] was used to detect changes in the gut microbiota-metabolite interactions in AD, laying a foundation for the early prevention and treatment of AD. The 16S rRNA gene in each sample from each microorganism was enriched through reversible solid-phase implantation, quantified by electrophoresis using an Agilent 2100 Bioanalyzer (Agilent, USA), and sequenced with an Illumina MiSeq sequencing system [23]. Meanwhile, we used LC-MS/MS to quantify the metabolites across groups and performed a targeted in-depth comparison of the blood metabolites from the APP/PS1 mice and C57 mice at 2 months of age. Considering the size of the dataset, the hierarchical structure of the metabolome, and the complex taxonomy of the microbiome, the metabolites and bacteria differed greatly at different levels. Thus, the use of multilevel strategies and multiple methods will simplify the complex data and ensure the accuracy and consistency of the results.

The purpose of this study is to establish a research strategy for determining the association between the microbiome and metabolome and to provide additional information on gut and brain interactions through techniques designed to analyze and mine data to identify potential therapeutic markers and to solve the complex problems associated with the pathological and pharmacological studies of AD. At the same time, this study provides new insights into the pathogenesis of AD from the perspective of microbe-metabolite correlations and may find that supplemental dietary metabolites delay AD or alleviate the pain caused by some diseases.

Thus, two levels of strategies, including microbial diversity analyses and metabolomics, were used to determine the underlying targets and mechanisms. In addition, the findings may lay the groundwork for the development of OMO as anti-AD drugs and health foods.

## 2. Materials and Methods

### 2.1. Preparation of the Animal Model

Thirty male APP/PS1 transgenic mice (2 months old) and 10 C57BL/6J male mice (2 months old) of the same age and genetic background were purchased from Beijing HFK Bioscience Co., Ltd. (certificate no. SCXK (Jing) 2014–0004); the mice had a mean body weight of 20 ± 5 g. The mice were housed in plastic cages in a temperature-controlled (25°C) colony room on a 12/12 h light/dark cycle. All animal experiments began at least 4 weeks after the animals arrived. The experimental mice were divided into three groups: the model group (APP/PS1 transgenic mice orally administered distilled water, *n* = 10), the normal group (C57BL/6J mice orally administered distilled water, *n* = 10), and the OMO administration group (APP/PS1 transgenic orally administered 100 mg/(kg·d) OMO, *n* = 10). Food and water were provided ad libitum. All experimental procedures were approved by the Center of Laboratory Animals of the Guangdong Institute of Microbiology.

### 2.2. Measurement of AD Parameters

The appearance and behavior of the animals were observed and recorded daily. The animal weights were measured every three days during the study. Following OMO treatment for 6 months, all animals were sacrificed; their eyeballs were removed, and their blood and brain tissue were collected. Routine serum parameters and cytokine levels [24] were measured, and the brains of the mice were dissected. Five brains from each group were fixed with a 4% paraformaldehyde solution, embedded in paraffin, and sectioned. Sections were stained with hematoxylin-eosin (H&E) or subjected to immunohistochemical staining and observed under a light microscope [17, 25]. All animal handling procedures and experiments were performed strictly in accordance with the recommendations of the Guide for the Care and Use of Laboratory Animals from the National Institutes of Health. The experimental protocol was approved by the Laboratory Animal Center and the Ethical Committee of the Guangdong Institute of Microbiology, Guangzhou, China. Each procedure described in our study was approved by the Laboratory Animal Center of the Guangdong Institute of Microbiology. This study utilized as few mice as possible.

### 2.3. 16S rRNA Analysis

Fecal specimens were acquired from the home cages of the mice and stored at −80°C until the 16S rRNA analysis. The QIAamp DNA Stool Mini Kit was used for extraction, and the extracted DNA was detected. The quality of the extracted DNA was determined by 0.8% agarose gel electrophoresis, and the DNA was quantified by a UV spectrophotometer. The 16S rRNA genes of the microbes were amplified with the following primers: forward 5′-ACTCCTACGGGAGGCAGCA-3′ and reverse 5-GGACTACHVGGGTWTCTAAT-3. Every amplified product was concentrated by SPRI, and an Agilent 2100 Bioanalyzer was used to quantify the samples subjected to electrophoresis. Every specimen was diluted to 1 × 10^9^ molecules/*μ*L in TE buffer and pooled into groups prior to the determination of DNA concentrations using a NanoDrop spectrophotometer. An Illumina MiSeq sequencing system was utilized to sequence 20 *μ*L of the pooled admixture, and the subsequent reads were analyzed.

### 2.4. Metabolomics Analysis

Serum samples were treated as described in a previous study [13]. The processed samples were loaded into a tube lining a sample vial and analyzed using LC-MS (Thermo Fisher Scientific, USA). The chromatographic column was an Acquity BEH C18 column (100 mm × 2.1 mm internal diameter, 1.7 *μ*m; Waters, Milford, USA). Mobile phase A was water (0.1% (v/v) formic acid), mobile phase B was acetonitrile (0.1% (v/v) formic acid), the flow rate was 0.40 mL/min, the injection volume was 3 *μ*L, and the column temperature was 45.0°C. Mass spectrometry data were acquired from 50 to 1,000 *m*/*z* with a resolution of 30,000. Metabolites were measured by a UPLC/LTQ Orbitrap mass spectrometer equipped with an electrospray interface (Thermo Fisher Scientific, USA).

### 2.5. Association Analysis

Alterations in the gut microbiome and serum metabolome of the normal and model groups were evaluated. The proportions of different metabolites were calculated by summing the levels of all metabolites (normalized by row, within each variable) of the corresponding types. The phyla were determined by summing all OTUs of the corresponding phyla. Then, a two-level strategy was adopted to determine the key association pairs. For the high-level association analysis, considering the size and complexity of the omics data, correlations between metabolite types and bacterial phyla were examined. The key “bacterial phylum-metabolite type” pairs were selected based on the results of (1) the regularized canonical correlation analysis (rCCA) [26] and Spearman correlation analysis on the abundance datasets [27], (2) relative abundance analysis of the phyla derived from the bacterial data, and (3) the Spearman correlation coefficient for each metabolite type and each bacterial phylum. Correlation networks were constructed based on the results from the correlation analyses, and all specific “bacterium-metabolite” association pairs were listed. Significant key pairs were selected for further analyses. Finally, the key pairs were validated in an OMO intervention study.

### 2.6. Statistical Analysis

All data presented in the figures are reported as the means ± standard deviations of at least three independent experiments. Significant differences between treatments were analyzed by the Kruskal–Wallis test/one-way analysis of variance (ANOVA) and set to *p* < 0.05 using the statistical packages in MATLAB (R2014a, MathWorks, USA), R (3.5.1), GraphPad Prism 7 (USA), and Cytoscape (3.6.1).

## 3. Results

### 3.1. Pathological Changes in the Normal and Model Groups

**Figure 1:**
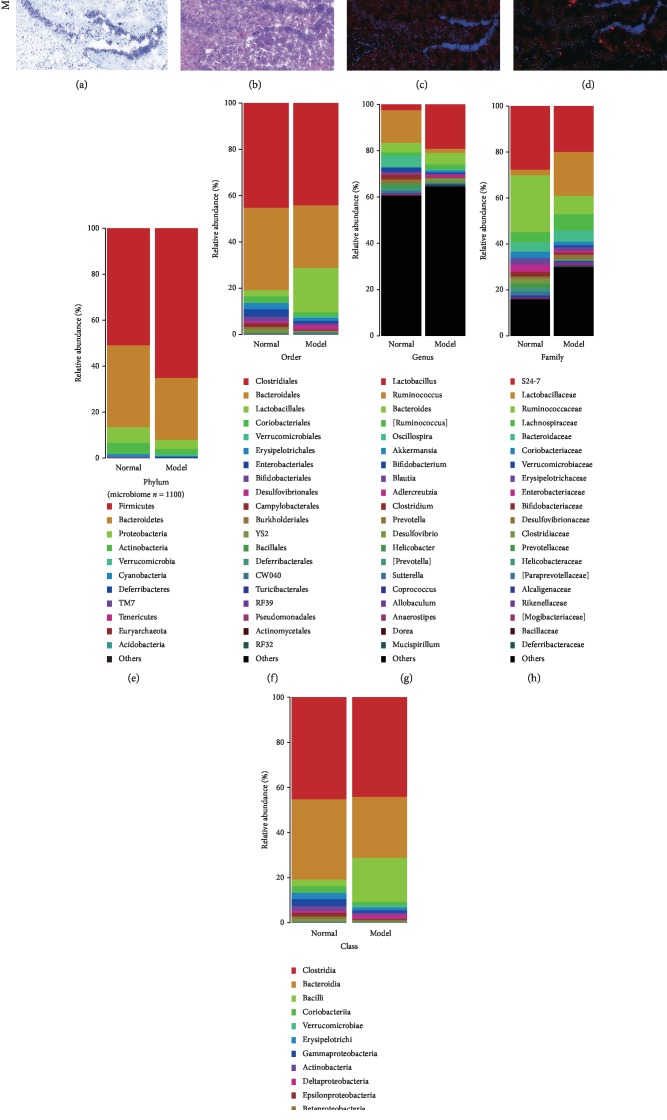
The gut microbiome composition and alterations across the AD study. (a)–(d) Histopathological changes in brain tissues. (e)–(i) Gut microbiome compositions. Normal represents the C57 group, and model denotes the APP/PS1 transgenic group; the management cycle was 6 months (*n* = 8). Values are presented as the means ± SDs of two independent experiments.

Nissl staining revealed more neurons (blue points) in the normal group than in the model group (Figure 1(a)). More hippocampal neurons were observed in the normal group than in the model group. H&E staining did not reveal obvious neuronal abnormalities in the hippocampus of the normal group (Figure 1(b)). The pyramidal cells in the CA1 region were arranged neatly and tightly, and no cells were lost. However, obvious histopathological damage to the hippocampus was observed in the model group. The layered pyramidal neuron structure was disintegrated, and neurons were lost in the CA1 region. Neurons with pyknotic nuclei and with shrunken or irregular shapes were also observed. Immunofluorescence staining showed an intense red color (p-tau and A*β*_1-42_) throughout the hippocampal region of the APP/PS1 double transgenic mice, while in the normal group, the RLU was obviously reduced. In CA1, the expression of p-tau and A*β*_1-42_ (red fluorescence) was barely detectable (Figures 1(c) and 1(d)).

### 3.2. Alterations in the Gut Microbiome and Metabolome

**Figure 2:**
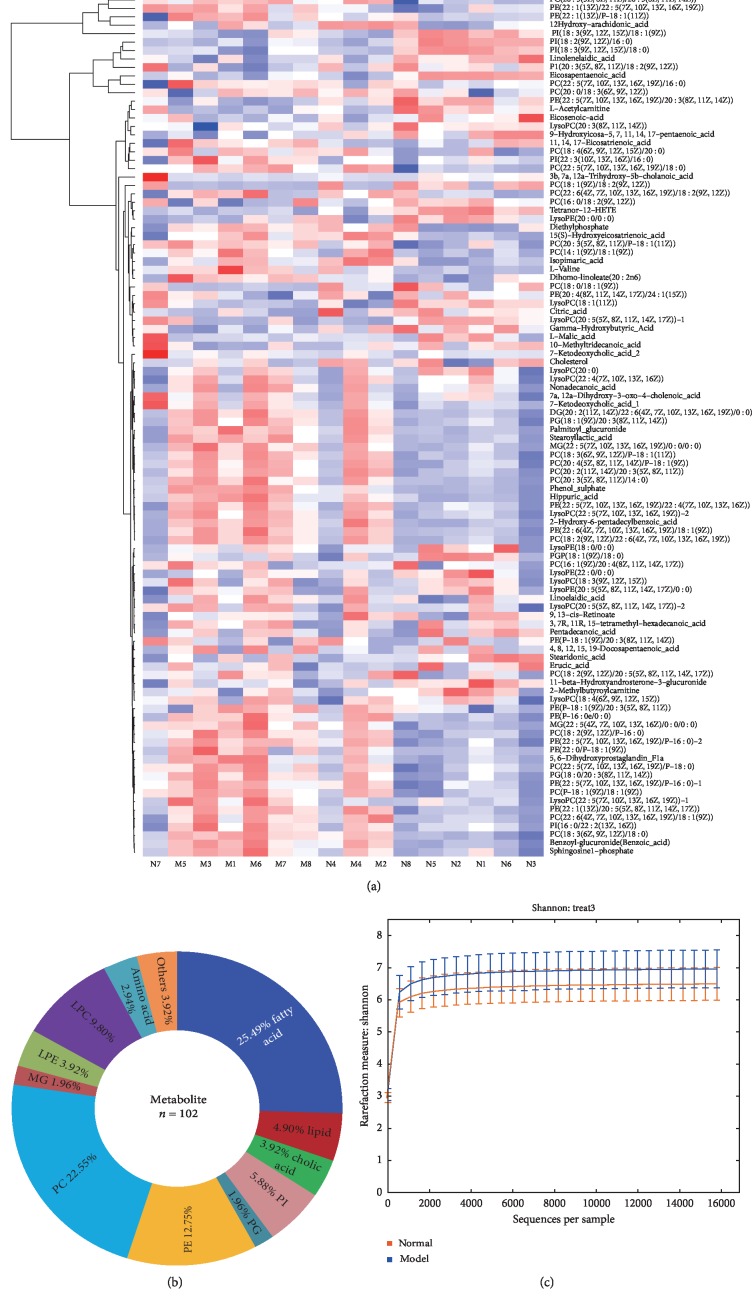
The serum metabolome composition and alterations across the AD study. (a) Heatmap of differential metabolic products between the normal group and model group. (b) Serum metabolome composition. (c) Shannon indexes of gut microbiome diversity. Normal represents the C57 group, and model denotes the APP/PS1 transgenic group; the management cycle was 6 months (*n* = 8). Values are presented as the means ± SDs of two independent experiments.

In this study, 11 bacterial phyla, 20 bacterial orders, 20 bacterial genera, 20 bacterial families, 20 bacterial classes (Figures 1(e) and 1(i)), and 102 different metabolites (11 metabolite types) were analyzed (Figures 2(a) and 2(b)). Firmicutes and Bacteroidetes were the predominant bacterial phyla found in the gut, while fatty acids and phosphatidylcholine (PC) were the predominant types of metabolites detected and accounted for 48.04% of all metabolites. The Shannon indexes (Figure 2(c)) revealed a higher gut microbiome diversity in the APP/PS1 transgenic mice than in the C57 mice. Alterations in the gut microbiome and metabolome were further evaluated by determining the relative abundances of bacterial phyla and metabolite types in the normal and model groups. As expected, both high and low abundance variables changed substantially. The Kruskal–Wallis test/one-way ANOVA revealed significant changes in eight metabolites (Figures 2(a) and 2(b)), namely, fatty acids, cholic acid, phosphatidylinositol (PI), phosphatidylglycerol (PG), phosphatidylethanolamine (PE), PC, amino acids, and lysophosphatidylcholine (LPC), and five bacterial phyla (Firmicutes, Bacteroidetes, Proteobacteria, Actinobacteria, and Cyanobacteria) (Figure 1(e)) between the two groups.

### 3.3. Correlation Analysis of the “Microbiota Phylum-Metabolite Type” Pairs

**Figure 3:**
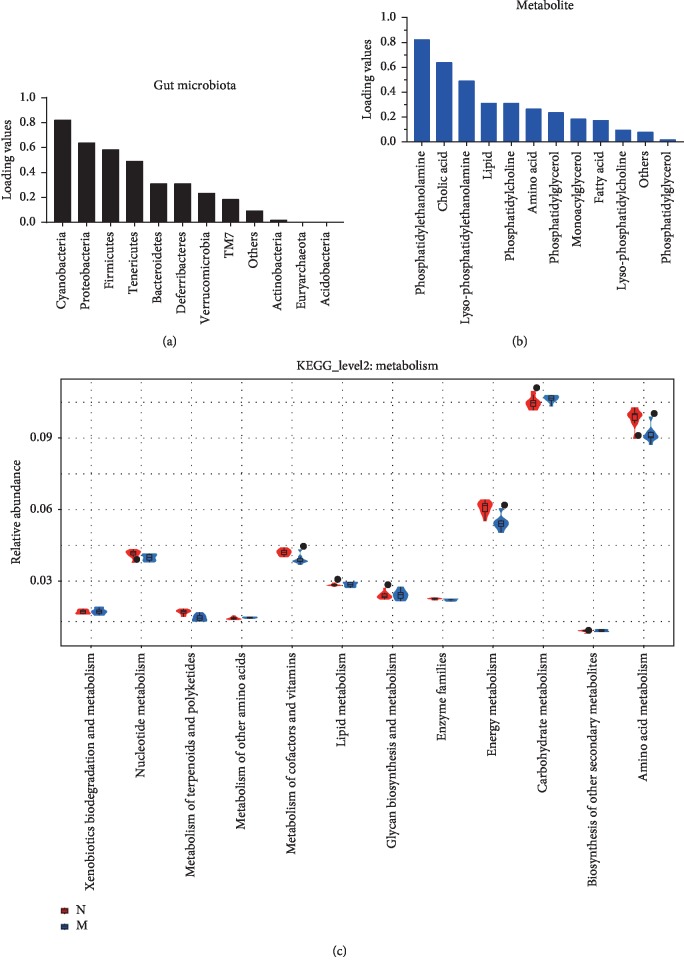
Results of the correlation analysis of the “microbial phylum-metabolite type” pairs in the APP/PS1 transgenic mice. (a) Loading values for every microbial phylum derived from the rCCA. (b) Loading values for every metabolite type derived from the rCCA. (c) Significant differences in metabolic functions were identified in the KEGG database. Normal represents the C57 group, and model denotes the APP/PS1 transgenic group.

The loading value (descending order) of each bacterial gate and metabolite type was calculated by rCCA, as shown in Figures 3(a) and 3(b). The loading values of eight bacterial phyla (Cyanobacteria, Proteobacteria, Firmicutes, Tenericutes, Bacteroidetes, Deferribacteres, Verrucomicrobia, and TM7) and ten metabolite types (PE, cholic acid, lysophosphatidylethanolamine, lipids, PC, amino acids, phosphatidylglycerol, monoacylglycerol, fatty acids, and LPC) were higher than the other parameters, indicating their greater contributions to the overall correlation between healthy and AD model mice.

**Figure 4:**
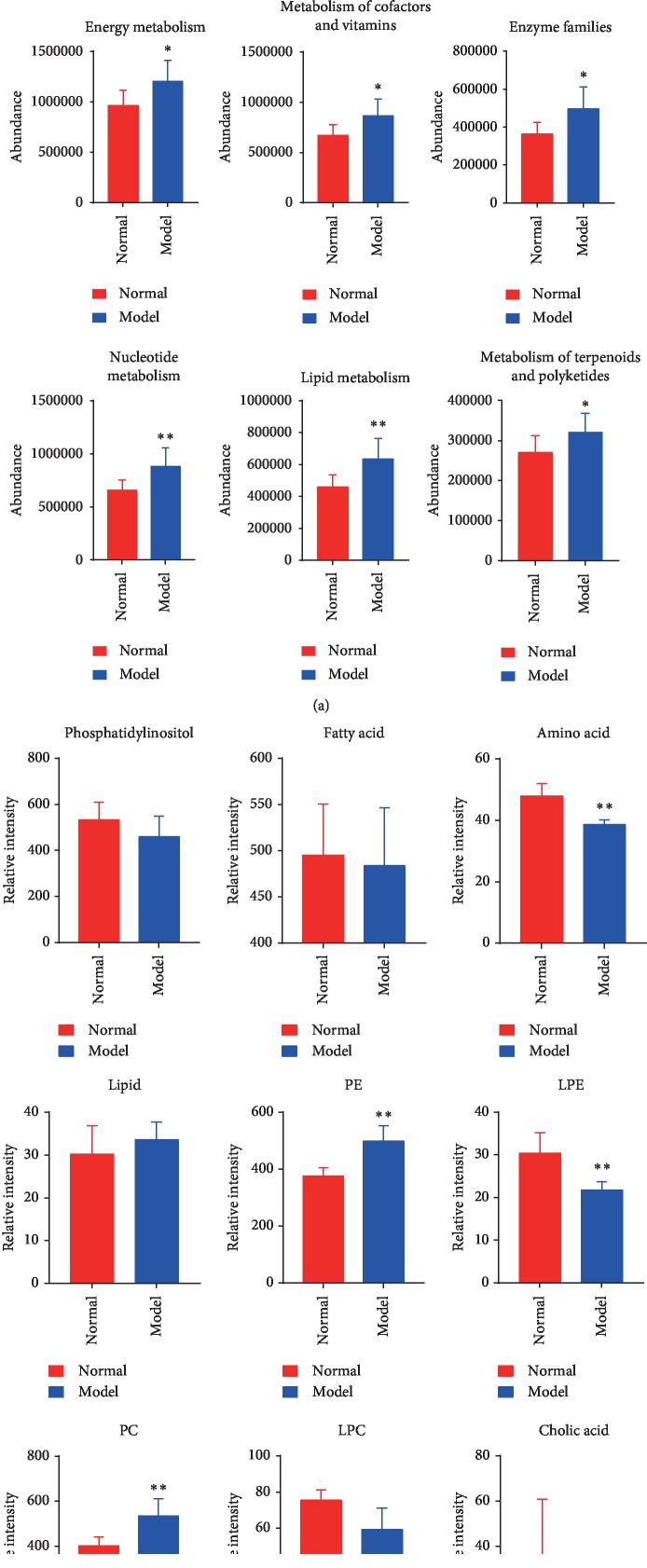
Differences in the predicted metabolic functions and levels of various metabolites in the two groups. Values are presented as the means ± SDs from six independent experiments. ^*∗*^*p* < 0.05 compared with the normal group; ^*∗∗*^*p* < 0.01 compared with the model group.

Phylogenetic Investigation of Communities by Reconstruction of Unobserved States (PICRUSt) was used to predict the Kyoto Encyclopedia of Genes and Genomes (KEGG) functions based on the predicted abundance distribution of each functional group, and the findings are shown in a violin plot. The 12 significantly differential metabolic functions that were identified are shown in Figure 3(c). The abscissa is the second functional group from KEGG, and the ordinate is the relative abundance of each functional group in each sample. Metabolic functions related to lipids, cofactors and vitamins, energy, amino acids, carbohydrates, and nucleotides were significantly altered between groups. The predicted metabolic functions are of great significance for the study of AD. As shown in Figures 4(a) and 4(b), the differentiation of the predicted metabolic functions (Figure 4(a)) and metabolite types (Figure 4(b)) varied between the two groups. The correlation coefficient for amino acid metabolic function and amino acid was −0.58 (*p* < 0.05, Figure 4), and these parameters were negatively correlated. This suggests that the amino acids in serum may be related to the metabolism and consumption of amino acids in other tissues. Compared with the control group, the amino acid, PE, LPE, and PC metabolites were significantly different in the model group (*p* < 0.05, Figure 4(b)).

**Figure 5:**
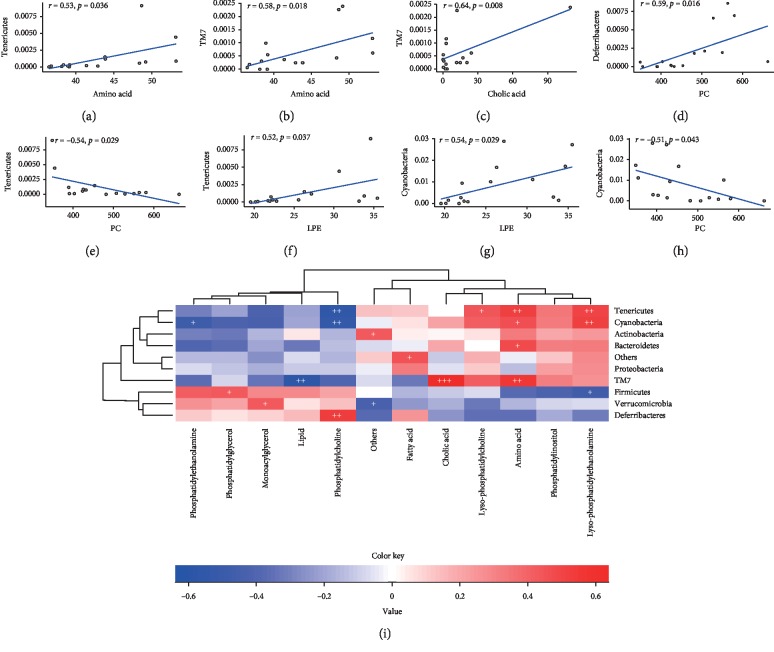
Results of the correlation analysis of the “microbial phylum-metabolite type” pairs in the AD study. (a−h) Scatter plots of the eight key high-level pairs. (i) Spearman correlation heatmap for each microbial phylum and each metabolite type (^+^*p* < 0.05, ^++^*p* < 0.01, ^+++^*p* < 0.001).

The classic Spearman correlation analysis was performed on abundance datasets to estimate the correlations between each bacterial phylum and every metabolite type (Figure 5). The top three metabolite types that correlated with the greatest number of bacterial phyla were amino acids, lysophosphatidylethanolamine, and PC, and the top three phyla that correlated with the greatest number of metabolite types were TM7, Tenericutes, and Cyanobacteria.

Based on these results, the variable abundance and biological significance, “lipids-TM7,” “amino acids-Tenericutes,” “lysophosphatidylethanolamine-Cyanobacteria,” and “PC-Cyanobacteria” were selected as key correlated pairs (Figure 5(i)). Figures 5(a)–5(h) show scatter plots of these pairs, and their correlation coefficients were 0.53, 0.58, 0.64, 0.59, 0.54, 0.52, 0.54, and −0.51 (*p* < 0.05).

### 3.4. Correlation Analysis of the “Microbiota-Metabolite” Pairs

**Figure 6:**
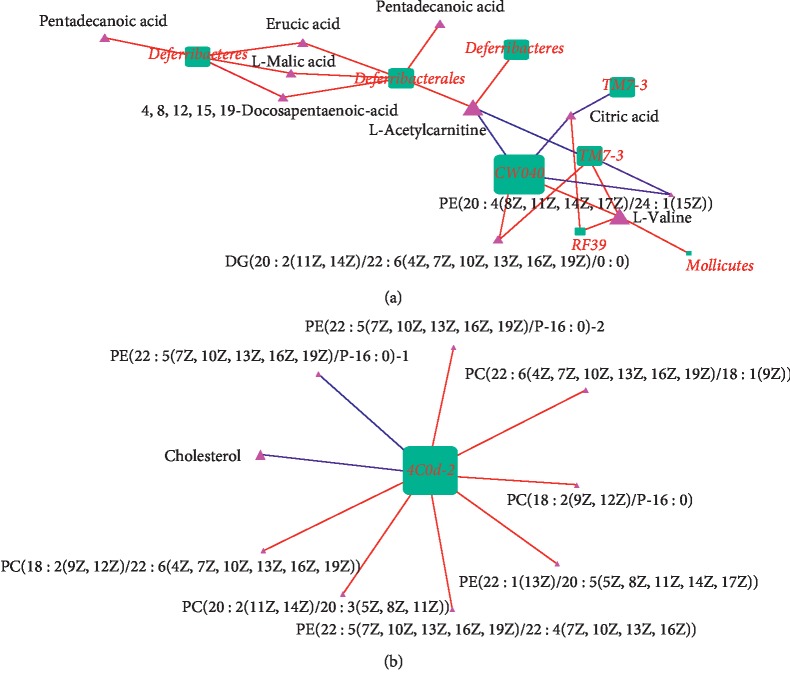
Significant pairs of correlated metabolites and genera/species in the TM7 and Deferribacteres phyla and the Cyanobacteria phylum. The red and blue lines indicate positive and negative correlations, respectively. Hub nodes with the greatest number of connections are highlighted. The green square represents the microbiome, and the purple triangle represents the metabolites.

In the normal group, 70 species and 267 genera were annotated in 4421 OTUs, and in the model group, 52 species and 289 genera were annotated in 4472 OTUs. A pairwise Spearman correlation analysis was conducted on the 111 bacteria (11 phyla, 20 orders, 20 genera, 20 families, 20 classes, and 20 species) and 102 metabolites. The results for 336 of the over 3300 pairs (9.82%) were significant after the FDR correction. Among these pairs, 187 (55.7%) were positively correlated, and 149 (44.3%) were negatively correlated.  Figure 6 shows the two correlation networks of bacterial genera/species (dot) and metabolites (triangle) with positive contributions (consistent correlation directions) to the five high-level key pairs. Four PCs, 6 PEs, 1 lipid, 4 fatty acids, 1 diacylglycerol (DG), 1 amino acid, 2 genera/species in TM7, 4 genera/species in Cyanobacteria, 2 genera/species in Tenericutes, and 1 genus in Deferribacteres were involved. The red and blue lines indicate positive and negative correlations, respectively. The size of each node is proportional to the number of lines connected to it, and the node shape indicates different bacterial classes and metabolite subtypes. Green represents bacteria, and red represents metabolites. The most extensively connected nodes are the key bacteria or metabolites. Four groups of correlation pairs were prominent, including (1) *4C0d-2* spp. in the phylum Cyanobacteria with 4 PEs, 4 PCs, and 1 lipid; (2) *CW040* spp. in the phylum TM7 with 1 citric acid, 2 amino acids, 1 DG, and 1 PE and TM7-3 spp. in the phylum TM7 with 1 DG and 1 amino acid; (3) *Deferribacterales* spp. in the phylum Deferribacteres with 4 fatty acids and 1 amino acid and the phylum Deferribacteres with 5 fatty acids and 1 amino acid; and (4) *RF39* spp. in the phylum Tenericutes with 1 amino acid and 1 citric acid. In Figure 6, two hub nodes (L-valine and L-acetylcarnitine) are highlighted, as they are all correlated with 6 bacteria, with comparable strengths.

First, both Cyanobacteria (*4C0d-2* spp.) and lipids are associated with brain development and disease. Cyanobacteria produce different complex lipid secondary metabolites [28] and possess strong biological properties and application value, such as antibacterial, antifungal, anticancer, antituberculosis, immunosuppressive, and anti-inflammatory properties. Over the past decade, AD has been shown to be closely related to changes in lipid levels or lipid metabolism. Thus, Cyanobacteria metabolize lipid products that may be associated with the progression of AD [29].

Second, in this report, TM7 was negatively correlated with lipids, and Cyanobacteria (*4C0d-2* spp.) was negatively correlated with PC. Among these metabolites, cholesterol [30], PC (22 : 6(4Z, 7Z, 10Z, 13Z, 16Z, 19Z)/18 : 1(9Z)), PC (20 : 2(11Z, 14Z)/20 : 3(5Z, 8Z, 11Z)), PC (18 : 2(9Z, 12Z)/22 : 6(4Z, 7Z, 10Z, 13Z, 16Z, 19Z)), and PC (18 : 2(9Z, 12Z)/P-16 : 0) are well-known dietary nutrients required for brain function [31] and have been linked to the morbidity and progression of AD. *Mollicutes* spp. may be associated with AD, although their pathogenic potential remains unclear [32]. Third, the close correlations between *TM7-3* spp. and L-valine, L-acetylcarnitine, PE (20 : 4(8Z, 11Z, 14Z, 17Z)/24 : 1(15Z)), and DG (20 : 2(11Z, 14Z)/22 : 6 (4Z, 7Z, 10Z, 13Z, 16Z, 19Z)/0 : 0) indicate the connection between the brain and gut, and the modulation of the activity of *TM7-3* spp. may be beneficial to human health [33–35].

Additionally, the links between pentadecanoic acid [36], L-malic acid [37], erucic acid, 4, 8, 12, 15, 19-docosapentaenoic acid, and Deferribacteres (*Deferribacterales* spp.) have been reported to affect the brain and influence neuropathy, thus providing circumstantial evidence of these association pairs in those reports.

Fourth, citric acid [38], pentadecanoic acid, erucic acid [39], and L-valine (the hub node with the most links in Figure 6) [40] were proposed as new therapeutic agents to regulate both neurobehaviors and the health of the gastrointestinal tract, although further studies are needed to define their possible roles in the gut-brain axis [41].

### 3.5. Validation of Correlated Pairs Using the OMO Intervention Study

Oligosaccharides are important factors related to the regulation of gut microbial composition and metabolite levels. An independent oligosaccharide intervention was conducted, and OMO served as the intervention for the model group. OMO originates from *M. officinalis*, which is one of the four great southern medicinal plants used as both a medicine and food, and it is processed by extraction and separation [21]. We conducted a validation study in the normal and model intervention groups and selected the same bacteria and metabolites for analysis with the same method to verify whether the key pairs identified in the two experiments were consistent. Spearman correlation analyses were performed for all the key pairs.

**Figure 7:**
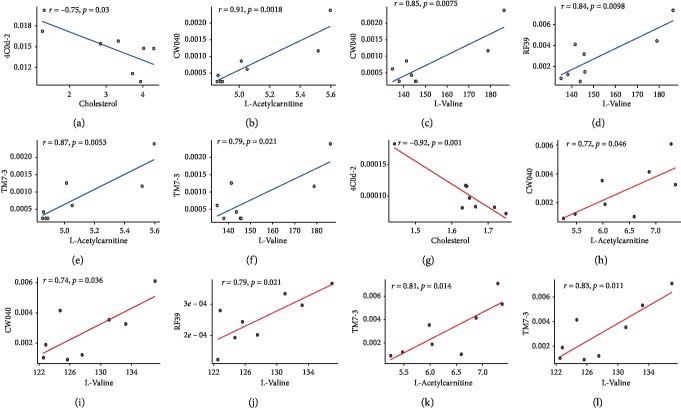
Spearman correlation coefficients and scatter plots of key correlation pairs in the normal group and the OMO intervention studies. (a)–(l) Scatter plots and correlation coefficients of the six representative pairs in the two studies. (a) and (e) 4C0d-2 spp.-cholesterol, (b) and (h) CW040 spp.-L-acetylcarnitine, (c) and (i) CW040 spp.-L-valine, (d) and (j) RF39 spp.-L-valine, (e) and (k) TM7-3 spp.-L-acetylcarnitine, and (f) and (l) TM7-3 spp-L-valine.

As shown in Figure 7, the correlation directions of the specific “microbiota-metabolite” pairs were largely consistent as well, although some of the bacteria shown in Figure 7 were not observed in the OMO intervention study. Figures 7(a)–7(l) display the detailed relationships of six representative “microbiota-metabolite” pairs in the two studies. These pairs are “*4C0d-2* spp.-cholesterol,” “*CW040* spp.-L-valine,” “*CW040* spp.-L-acetylcarnitine,” “*RF39* spp.-L-valine,” “*TM7-3* spp.-L-valine,” and “*TM7-3* spp.-L-acetylcarnitine.” The correlation directions of “4C0d-2 spp.-cholesterol” (*r* = −0.75 and −0.92 in the normal and OMO intervention groups, respectively), “*CW040* spp.-L-acetylcarnitine” (*r* = 0.91 and 0.72), “*CW040* spp.-L-valine” (*r* = 0.85 and 0.74), “RF39 spp.-L-valine” (*r* = 0.84 and 0.79), “TM7 spp.-L-acetylcarnitine” (*r* = 0.87 and 0.81), and “TM7 spp.-L-valine” (*r* = 0.79 and 0.83) were all consistent between the 2 studies.

The relative abundance of *CW040* [42] and *TM7-3* (TM7) was increased in the model group, and the abundance of *Deferribacterales* (Deferribacteres) was decreased (Figures 1(e), 1(f), and 1(i)). The level of the metabolite L-acetylcarnitine [43] was increased in the OMO group, and the levels of cholesterol [44] and L-valine were decreased (Figure 2(a) and Figures 7(a)–7(l)). The pathological changes observed were alleviated in the OMO intervention experiment [13].

Collectively, the association directions of the key correlation pairs were largely consistent in the two studies. These microorganisms and metabolites may be potential targets for the treatment of AD. However, the intestinal microorganisms and metabolites in animal and human samples also differ, and these “microbiome-metabolite” pairs are key to specific change rules that need to be further studied. Thus, we should exercise caution when applying the findings from animal experiments to humans.

### 3.6. Possible Routes Connecting the Gut and Metabolites

We used both microbial and metabolomic analytical strategies, and “Tenericutes-amino acid,” “Tenericutes-PC,” “Cyanobacteria-LPE,” “Cyanobacteria-PC,” and “TM7-cholic acid” were identified as probable key endpoints in the pathway that connects the brain to the gut.

The two-level analysis of microorganisms and metabolites was an effective method to simplify complex data and effectively identify the correlations. However, this strategy may ignore less statistically significant but more biologically relevant factors, such as the types of microphytes and metabolites that are not selected as key pairs but play an important role in the gut-brain interaction.

As shown in Figure 5(i), LPE was strongly and positively correlated with not only Tenericutes but also Cyanobacteria and Firmicutes. In addition to its correlation with LPE, Tenericutes was also closely correlated with amino acids, LPC, and PC, which are important for synaptic activity and protein synthesis. Another example of a combination with low statistical significance but high biological significance was the “microbiota-metabolite” pairs; the metabolites were not correlated with five key high-level microbial phyla but may also improve our understanding of the microbial-gut-brain axis. These pairs included species in the orders Clostridiales/Lactobacillales/Erysipelotrichales/Bacillales/Turicibacterales and lipids; species in the orders Bacteroidales/Flavobacteriales and PC; species in the orders Enterobacteriales/Desulfovibrionales/Campylobacterales/Burkholderiales/Pseudomonadales/Hydrogenophilales/Chromatiales/HOC36/Desulfarculales and fatty acids; Verrucomicrobiales and decanoic acid; and species in the orders Coriobacteriales/Bifidobacteriales/Actinomycetales and decanoic acid. Although these microbes and metabolites exert important effects on brain and intestinal health [12, 45], the overall performance of each metabolite type or phylum was not statistically significant. Therefore, the biological significance of a low-level correlation without a hierarchical relationship between the low-level correlation and high-level key correlation should be carefully evaluated when applying the two-level strategy to analyze the microbiota and metabolites.

## 4. Discussion and Conclusion

As China becomes an aging society, the increasing incidence of AD will be a heavy burden for society and for medical care, so new treatments and potential drugs are urgently needed. The evidence shows that the pathological features of AD are related to microbial infection, so the gut microbiota is considered an important factor determining the progress of AD. Previous studies have shown that OMO can improve the symptoms of animal models of AD, which is related to regulation of the diversity of the gut microbiota and metabolic components. The two-level strategy confirmed the relevance between AD disease and key microbiota-metabolite pairs, and the OMO intervention studies further validated their relationship.

Lipids are a group of metabolites with multiple biological functions that are closely related to various metabolites [46]. Based on accumulating evidence, the gut microbiota, as an environmental factor, modulates the processing, absorption, metabolism, storage, clearance, and other systemic functions of host lipids in the elderly [47–49]. Fatty acids are a group of carboxylic acids with different numbers of aliphatic chains that are either saturated or unsaturated. A large number of studies have revealed the remarkable effects of free fatty acid metabolism on brain circulation, structure, and function at different ages [50]. According to some studies [51, 52], old mice display significantly increased levels of both amino acids and fatty acids, which are closely associated with AD biomarkers, compared to young mice. In the intestinal microbiome analyses, the Firmicutes/Bacteroidetes ratio and alpha diversity were increased in older mice.

The gut microbiota synthesizes a variety of nutrients and essential amino acids, representing a potential mechanism for regulation of amino acid homeostasis [53]. In recent years, close relations between the human gut microbiome, the physiological aspects of its metabolites and hosts, and the important pathological and physiological dimensions of diseases have been identified [53]. Amino acids are involved in many cellular metabolic and signaling pathways, and the effects of changes in amino acid metabolism are far-reaching in patients with AD [54].

PC and PE are the most abundant phospholipids in mammalian cell membranes [55]. Decreased levels of PC, which lead to decreased numbers of synapses in the brain, are a characteristic of memory disorders in patients with early AD [31]. PE [56] has a variety of cellular functions, including serving as a precursor of PC and as an important posttranslational substrate, affecting membrane topologies and facilitating cell and organelle membrane fusion, oxidative phosphorylation, mitochondrial biogenesis, and autophagy. The importance of PE metabolism in mammalian health has recently been revealed based on its associations with AD, Parkinson's disease (PD), nonalcoholic liver disease, and some pathogenic microorganisms [57]. Dietary nutrients containing PC and PE may be involved in the development of AD. The nutrients, the gut microbiota, and host participation in these metabolic pathways may be new targets for the prevention and treatment of AD.

Firmicutes and Bacteroidetes are known to be the dominant phyla in the gut and are involved in many brain functions [58]. They are also reported to be the phyla that display the greatest variation across their life cycles, independent of gender, age, and nationality [59]. However, clear relationships between these bacteria and the progression of AD have not been determined. Tenericutes [60] are unique and rare bacteria with no cell walls, and they survive as typical parasites or eukaryotic host symbionts. Environmental 16S rDNA investigations have identified several branches of Tenericutes in different environments, suggesting that these Tenericutes may represent free-living microorganisms that are independent of the host. The diversity of the fecal microflora in patients with polycystic ovary syndrome was low, and their phylogenetic components were altered. When rare taxa were observed, the relative abundance of the bacteria from the phylum Tenericutes was significantly reduced [61] and was correlated with reproductive parameters.

Cyanobacteria are microorganisms found in marine, freshwater, and terrestrial environments worldwide. Under favorable conditions, they form a large number of populations that harm human and animal health [58]. Toxins produced by Cyanobacteria may cause some form of nerve damage, and many of their effects are consistent with neurodegeneration [62]. Recently, a neurotoxin produced by Cyanobacteria that can cause neurodegenerative disease in humans was detected in the brain and cerebrospinal fluid of a patient with AD [63].

The key roles of the microbiome-gut-brain axis in the human lifespan [64, 65] and the patterns of characteristic changes in the gut microbiota [66–69] are actively being studied; furthermore, the metabolome has been extensively studied separately in the context of AD [70–73]. The main contribution of this report is that it is a groundbreaking joint study that uses two histological platforms to study the gut microbiome and brain metabolome of AD models. However, the intestinal microorganisms and metabolites of animal and human samples also differ. The associations identified in transgenic animals must be carefully verified in humans and further evaluated under different pathological conditions. The subsequent key pairs should be validated by further sequencing. More information can be obtained from the blood and fecal metabolome and microbiome, further confirming the connection between the blood metabolome and the intestinal microbiome and thus further establishing a systematic and integral network connecting the brain and gut.

The second contribution of this report is the design of key and relevant screening strategies. Considering the size of the dataset, the complex taxonomy of the microbiome, the hierarchical structure of the metabolome, and the diverse performance of metabolites and bacteria at different levels, as well as the unpredictable cross-correlations within the metabolome and microbiome, we conducted a two-level correlation analysis using data from the normal and AD model groups. In the first level, the univariate and multivariate correlation analyses and functional analysis were combined to select the key pairs. In the second level, we focused on pairing with a positive contribution to high-level pairing. The multilevel strategy and multiple methods simplified the complex data and ensured the accuracy and consistency of the results.

In addition to the emphasis on the correlated bacterial-metabolic pairs, other allelopathic correlations are worthy of further study. However, due to the limited size of our sample and the limitations of the APP transgenic mouse model, we may have missed some relationships observed in different types of AD.

We report a two-level association strategy for the analysis of the gut microbiome and metabolome in samples from the two groups. The high-level “bacterial phylum-metabolite type” and the lower-level “microbe-metabolite” correlation pairs were identified by an rCCA and Spearman correlation analysis. Correlation networks were constructed based on the results from the correlation analyses, and all the specific “microbiota-metabolite” association pairs were listed. Six associations of specific “microbiota-metabolite” pairs were further identified. The key relevant pairs were verified by an independent oligosaccharide intervention study, and the gut microbiome and serum metabolome of the OMO intervention group were similar to those of the normal group in the overall adjusted models.

However, current studies have shown that intestinal microorganisms and metabolites differ in both animal and human samples of AD at different stages, and it is not clear whether the key “microbe-metabolite” pairs have biological effects at other stages. We selected only one point in the present study, but the regular changes in “microbiome-metabolite” pairs at each stage will be further studied in the future.

Therefore, this study is a good start, and our findings offer new and early insights into the interactions between the blood metabolome and gut microbiome, indicating new potential pathways that connect the gut and brain and improving our understanding of the microbial-gut-brain axis. In future studies, we must further harness metagenomics to identify 1-2 bacterial strain and metabolite pairs that will be verified in animal experiments.

## Data Availability

The data used to support the findings of this study are available from the corresponding author upon request.

## Abbreviations

AD: Alzheimer's disease
OMO: Oligosaccharides isolated from *Morinda officinalis*
APP/PS1: APPswe/PS1dE9
rCCA: Regularized canonical correlation analysis
ANOVA: Analysis of variance
PC: Phosphatidylcholine
PI: Phosphatidylinositol
PG: Phosphatidylglycerol
PE: Phosphatidylethanolamine
LPC: Lysophosphatidylcholine
PICRUSt: Phylogenetic Investigation of Communities by Reconstruction of Unobserved States
KEGG: Kyoto Encyclopedia of Genes and Genomes.
